# *In silico* analysis to identify novel ceRNA regulatory axes associated with gallbladder cancer

**DOI:** 10.3389/fgene.2023.1107614

**Published:** 2023-02-16

**Authors:** Neeraj Saklani, Varnit Chauhan, Javed Akhtar, Santosh Kumar Upadhyay, Ravi Sirdeshmukh, Poonam Gautam

**Affiliations:** ^1^ Laboratory of Molecular Oncology, ICMR- National Institute of Pathology, New Delhi, India; ^2^ Department of Biotechnology, Gautam Buddha University, Greater Noida, Uttar Pradesh, India; ^3^ Department of Biotechnology, Sir J. C. Bose Technical Campus, Bhimtal, Kumaun University, Nainital, Uttarakhand, India; ^4^ Manipal Academy of Higher Education (MAHE), Manipal, India; ^5^ Institute of Bioinformatics, International Tech Park, Bangalore, India

**Keywords:** gallbladder cancer, ceRNA regulatory network, lncRNA-miRNA-mRNA regulatory axes, P53 signaling pathway, therapeutic applications

## Abstract

Competitive endogenous RNA (ceRNA) networks are reported to play a crucial role in regulating cancer-associated genes. Identification of novel ceRNA networks in gallbladder cancer (GBC) may improve the understanding of its pathogenesis and might yield useful leads on potential therapeutic targets for GBC. For this, a literature survey was done to identify differentially expressed lncRNAs (DELs), miRNAs (DEMs), mRNAs (DEGs) and proteins (DEPs) in GBC. Ingenuity pathway analysis (IPA) using DEMs, DEGs and DEPs in GBC identified 242 experimentally observed miRNA-mRNA interactions with 183 miRNA targets, of these 9 (CDX2, MTDH, TAGLN, TOP2A, TSPAN8, EZH2, TAGLN2, LMNB1, and PTMA) were reported at both mRNA and protein levels. Pathway analysis of 183 targets revealed p53 signaling among the top pathway. Protein-protein interaction (PPI) analysis of 183 targets using the STRING database and cytoHubba plug-in of Cytoscape software revealed 5 hub molecules, of which 3 of them (TP53, CCND1 and CTNNB1) were associated with the p53 signaling pathway. Further, using Diana tools and Cytoscape software, novel lncRNA-miRNA-mRNA networks regulating the expression of TP53, CCND1, CTNNB1, CDX2, MTDH, TOP2A, TSPAN8, EZH2, TAGLN2, LMNB1, and PTMA were constructed. These regulatory networks may be experimentally validated in GBC and explored for therapeutic applications.

## 1 Introduction

Gallbladder Cancer (GBC) is among the most common malignant tumor of the gastrointestinal system. The only effective treatment is complete surgical resection for this cancer which can be given to only about 10% of patients because this cancer is usually diagnosed at advanced stages, however, recurrence rates after surgical resections are high (Zhu et al., 2010). GBC patients have an overall median survival of 19 months and a 5-year survival rate of 28.8% (Zhu et al., 2020) despite standard treatment, including chemotherapy, radiotherapy and targeted therapy. Therefore, it is necessary to understand the molecular mechanism associated with GBC and identify novel targets for therapeutic applications in GBC.

MicroRNAs (miRNAs) are a class of small non-coding RNAs (ncRNAs) of approximately ∼22 nucleotides long. Their mRNA targets are based on limited sequence complementarity between the miRNAs seed region (first 2–7 nucleotides from the 5′ end) and regions in the 3′ untranslated region (3’ -UTR) of the mRNA. miRNAs play a key role in the negative regulation of target genes and thus change the cellular environment. Nearly 60% of the total known mRNAs are regulated by miRNAs (Friedman et al., 2008). Another group of ncRNA—long non-coding RNAs (lncRNAs), which are >200 nucleotides in length, also affect mRNA stability (Sebastian-delaCruz et al., 2021). LncRNAs containing a ‘miRNA response element’ can compete with other RNAs and are believed to act as competing endogenous RNAs (ceRNAs). LncRNA-mediated ceRNA regulatory network, namely, lncRNA/miRNA/mRNA axis, is important in promoting tumorigenesis and can potentially serve as a handle to identify key therapeutic targets (Xu et al., 2022).

There are several high-throughput studies available in GBC to identify differentially expressed mRNAs (DEGs) and proteins (DEPs) (Kim et al., 2008; Miller et al., 2009; Huang et al., 2014; Wang et al., 2020a). Knowledge of the non-coding RNAs regulating the expression of these cancer-associated genes/proteins and the corresponding regulatory networks would be important to understand the pathogenesis of GBC and for therapeutic applications. Various groups have analyzed the differential expression of miRNAs (DEMs) in tissue (Letelier et al., 2014; Zhou et al., 2014; Goeppert et al., 2019), serum/plasma extracellular vesicles (Ueta et al., 2021; Yang et al., 2022) using high-throughput studies in GBC. Similarly, other researchers have analyzed differentially expressed lncRNA (DELs) using high-throughput studies (Ma et al., 2016; Wang et al., 2017a; Wu et al., 2017), however, the majority of the studies on lncRNA in GBC are targeted studies. Few of these studies revealed the “lncRNA-miRNA-mRNA network” in GBC in a targeted manner (Wang et al., 2016a; Hu et al., 2019; Yang et al., 2020; Zhang et al., 2020). Another study has constructed a ceRNA network using DELs, DEGs data and predicted miRNAs based on the DEGs from a single GBC dataset (GSE76633) (Kong et al., 2019a). In view of the availability of various targeted and high-throughput studies at lncRNA, miRNA, mRNA, and protein levels in GBC, the construction of a “ceRNA regulatory network” (lncRNA/miRNA/mRNA axis) based on multiple experimental datasets would be highly relevant.

We aimed to uncover the potential ceRNA regulatory networks regulating cancer-associated processes involved in the development of GBC. The present study applied multi-omics datasets available in GBC, including high-throughput GBC-proteomics data from our lab (Akhtar et al., 2023), and RNA interaction databases (predicted/experimentally verified) to identify novel lncRNA-miRNA-mRNA regulatory networks in GBC which may be explored for their therapeutic applications in GBC.

## 2 Methodology

### 2.1 Data collection

The literature search was performed and studies with high-throughput expression data in GBC were included for miRNA and mRNA. As the detection of proteins is low in comparison to mRNAs or miRNAs, due to technical limitations, here, both high-throughput studies as well as targeted studies in GBC were used to achieve a comprehensive DEP dataset. The high-throughput proteomic studies include the data from our lab (Akhtar et al., 2023). For lncRNA, only targeted studies were used as their interactions with miRNA are well-annotated.

### 2.2 miRNA-mRNA regulatory axis of gallbladder cancer

Non-redundant lists of DEMs and DEGs were imported into QIAGEN IPA (QIAGEN Inc., https://digitalinsights.qiagen.com/IPA) (Krämer et al., 2014) and identified the miRNA-mRNA interactions. Similarly, non-redundant lists of DEMs and DEPs were imported into IPA and identified the miRNA-mRNA interactions. miR IDs (for DEMs) and gene symbols (for DEGs and DEPs) were used for IPA analysis.

The expression of lncRNA/miRNA/mRNA/protein was considered “Up” or “Down” based on the expression trend in >50% of the studies. The ones showing an opposite expression in equal no. of studies (50%) were not considered for any further analysis. Similarly, in the case of multiple transcripts of a gene the expression was considered “Up” or “Down” based on the expression trend in >50% of the transcripts.

For IPA analysis, an expression pairing filter was applied to include miRNA-mRNA pairs which are showing an opposite correlation in expressions (miRNA-Up/mRNA-Down; miRNA-Down/mRNA-Up). The confidence level filter was used to include only those interactions which were ‘experimentally observed’ or ‘predicted with high confidence’ [the cumulative weighted context score (or “CWCS”) as defined by TargetScan is −0.4 or lower]. The datasets from miRNA-mRNA interactions from DEGs and DEPs were integrated and obtained a non-redundant list of miRNA-mRNA interactions. “Experimentally observed” miRNA-mRNA interactions (from the non-redundant list) were selected and miRNA regulatory network was constructed using Cytoscape software v3.9.1 (https://cytoscape.org/) (Shannon et al., 2003).

### 2.3 Functional enrichment analysis

The Search Tool for Retrieval of Interacting Genes database (STRING version 11.5; https://string-db.org) (Szklarczyk et al., 2019) is an online database and tool that can build protein-protein interaction (PPI) network based on known and predicted interactions. Gene ontology analysis for “experimentally observed” miRNA targets was performed through STRING (cellular components and biological processes) and IPA (molecular and cellular functions and canonical pathways).

### 2.4 Protein-protein interaction analysis

The PPI of the “experimentally observed” miRNA targets were analyzed using STRING 11.5, [Organism: *Homo sapiens* and PPI score was set as 0.9 (highest confidence)]. The network was visualized by cytoscape v3.9.1. Cytohubba, a plugin of cytoscape software, was used to identify the hub genes of the PPI network (Chin et al., 2014). The intersection of the top 10 nodes ranked by degree, closeness, betweenness and bottleneck centrality were considered hub genes.

### 2.5 ceRNA regulatory network construction

DELs (targeted studies) and DEMs (miRNAs associated with hub molecules among the top pathway and the targets reported at both mRNA and protein levels) were used to screen the experimentally validated interaction between them by DIANA-LncBase v3 (https://diana.e-ce.uth.gr/lncbasev3) (Karagkouni et al., 2020). Both the subunits of miRNA, i.e., “-3p” and “-5p” were considered for finding associated lncRNAs in those cases where the subunits were not specified. Then, lncRNA-miRNA and miRNA-mRNA co-expression pairs (positive relation) were then used to construct ceRNA interaction networks (lncRNA-miRNA-mRNA). The networks were visualized using Cytoscape. Another database, mirTarBase (mirtarbase.cuhk.edu.cn), containing more than three hundred and sixty thousand miRNA-mRNA interactions was then used to categorize the miRNA-mRNA interactions with strong evidence (Reporter assay/Western blot/qPCR) or less strong evidence (Microarray, NGS, pSILAC, CLIP-Seq and others).

## 3 Results

**FIGURE 1:**
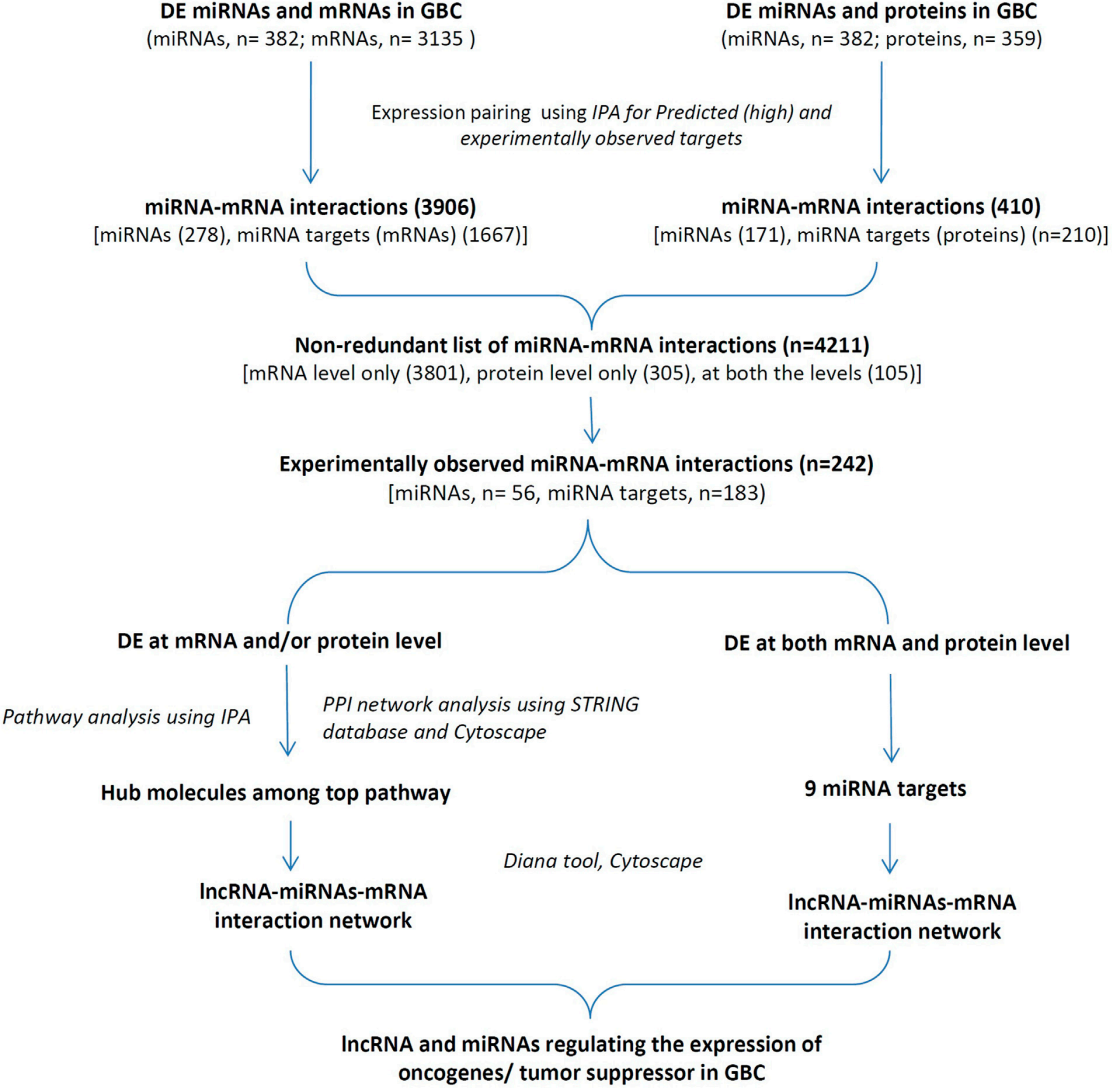
The overall workflow of the study. DE miRNAs, mRNAs, proteins and lncRNAs in GBC based on a literature survey were used for the study. DE, Differentially expressed; GBC, Gallbladder Cancer; IPA, Ingenuity pathway Analysis; PPI, protein-protein interaction.

The study design and workflow of the study is shown in Figure 1. ‘Tissue-based’ datasets (DELs, DEMs, DEGs, and DEPs) were used from high-throughput and/or targeted studies and performed IPA analysis for miRNA-mRNA interactions (predicted and experimentally observed) using DEMs and DEGs or DEPs. The “experimentally observed targets” were further used for associated canonical pathways and PPI analysis to identify hub molecules. In addition, miRNA targets DE at both mRNA and protein levels with a positive correlation in expression were also analyzed. Finally, lncRNA-miRNA-mRNA regulatory networks were constructed for the selected targets possibly functional in GBC.

### 3.1 DEM, DEG, DEP, and DEL dataset

Literature search revealed three high-throughput studies on tissue miRNA analysis in GBC (Letelier et al., 2014; Zhou et al., 2014; Goeppert et al., 2019). A total of 382 DEMs (non-redundant list) were obtained and shown in Supplementary Table S1. A total of seven high-throughput studies were found analyzing the expression of mRNAs in tissues. A total of 3,135 DEGs (non-redundant list) were obtained and are shown in Supplementary Table S2. Both high-throughput and targeted studies on the differential expression of proteins in GBC tissues were used for the analysis. Data on DEPs from our lab (Akhtar et al., 2023) was also included. A total of 359 DEPs (non-redundant list) were obtained and are shown in Supplementary Table S3. For lncRNAs, 45 targeted studies representing expressions of 43 different lncRNAs (non-redundant list) were found and is shown in Supplementary Table S4.

### 3.2 miRNA-mRNA regulatory axis of GBC

First, non-redundant lists of DE miRNAs (*n* = 382) (miRNA IDs) and DE mRNAs (*n* = 3,135) (gene symbol) were imported into IPA, out of which 372 miRNAs and 2,996 mRNAs were mapped. Inverse expression pairing resulted in a total of 3,906 miRNA and mRNA interactions “Dataset 1” (with high prediction and/or experimentally observed) that includes 278 miRNAs and 1,667 miRNA targets (mRNAs).

Similarly, non-redundant lists of DE miRNAs (*n* = 382) (miRNA IDs) and DE proteins (*n* = 359) (gene symbol) were imported into IPA, out of which 372 miRNAs and 356 mRNAs were mapped. Expression pairing resulted in a total of 410 miRNA and mRNA interactions “Dataset 2” (with high prediction and/or experimentally observed) that includes 171 miRNAs and 210 miRNA targets (proteins).

**FIGURE 2:**
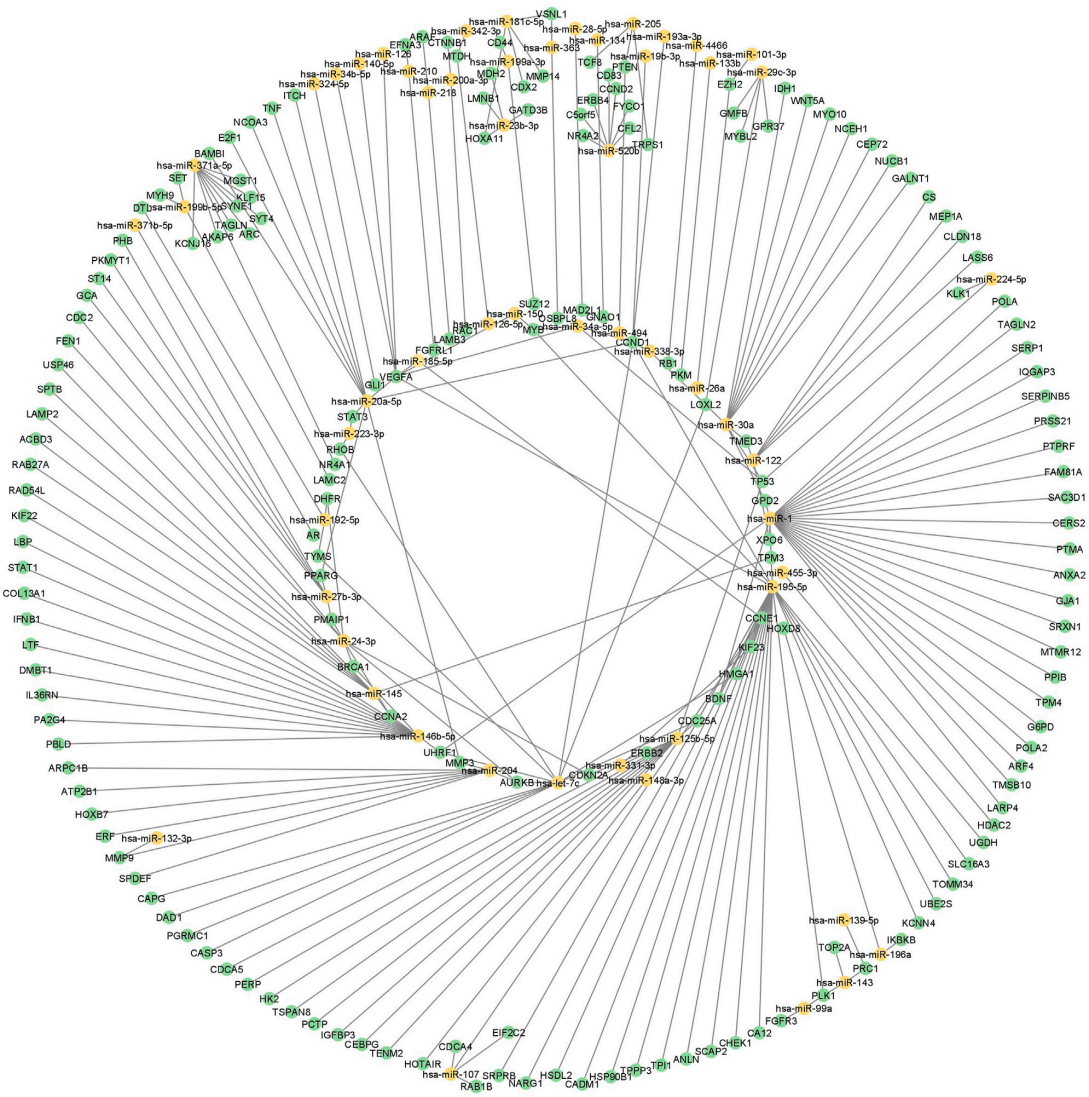
miRNA regulatory network in GBC. miRNA-mRNA regulatory network using DE miRNAs, mRNAs and proteins in GBC. Yellow color represents miRNAs and green represents mRNA or protein. GBC, Gallbladder Cancer; DE, Differentially expressed.

**FIGURE 3:**
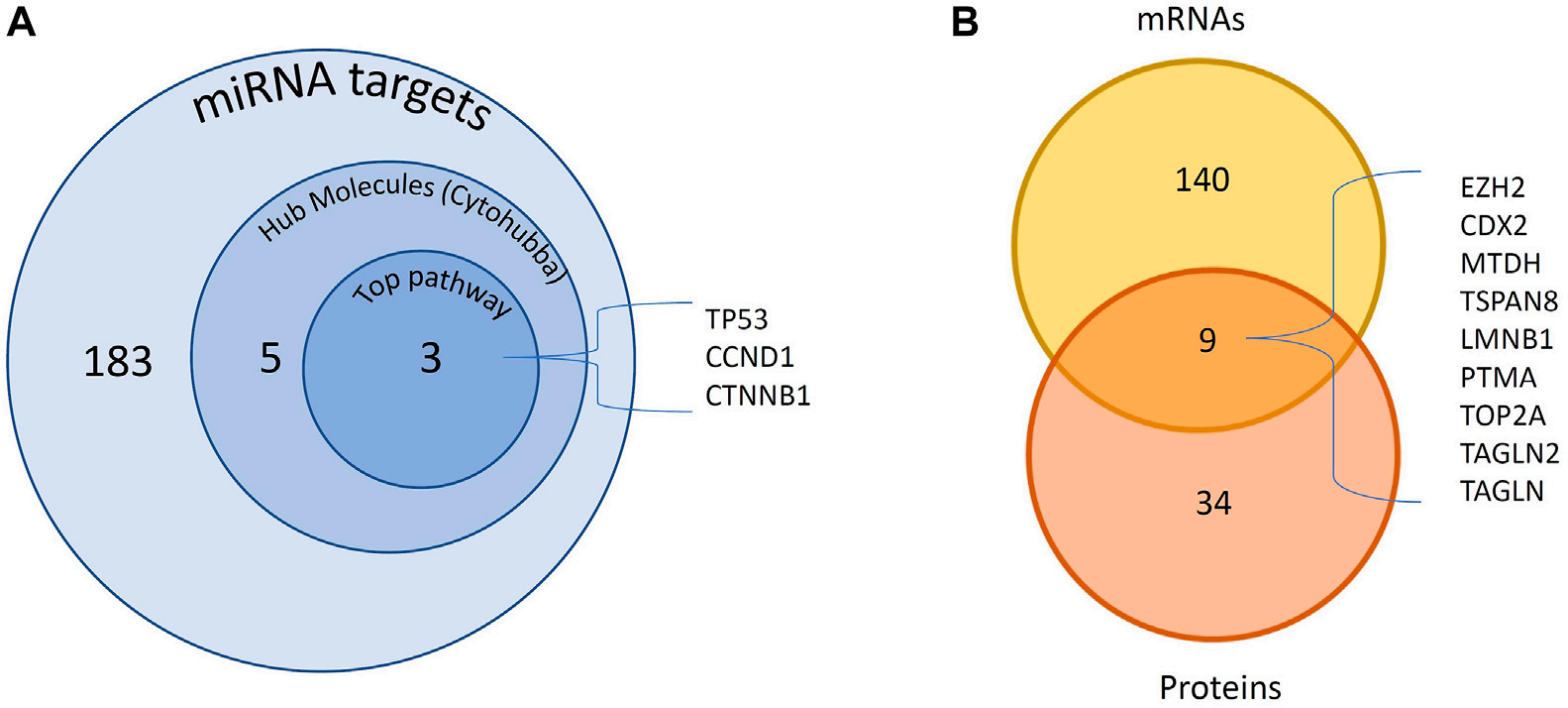
DE miRNAs and their targets DE in GBC. A total of 183 experimentally observed targets were identified. **(A)** PPI network analysis of 183 proteins showed 5 hub molecules. Pathway analysis showed p53 signaling among the top pathway which includes 3 of the hub molecules. **(B)** We found 9 miRNA targets reported to be DE at both mRNA and protein levels. DE- Differentially expressed; GBC, Gallbladder Cancer; PPI- protein-protein interaction.

The above two datasets were integrated and obtained a non-redundant list of 4,211 miRNA-mRNA interactions (Supplementary Table S5). This includes 242 interactions with “experimentally observed targets” (Figure 2) and 3,969 interactions with targets “predicted with high confidence”. These 242 interactions include 183 targets (Figure 3, Supplementary Table S6) and were used for gene ontology, pathway, and protein-protein interaction (PPI) network analysis. Out of 242, a total of 11 interactions include 9 targets (CDX2, MTDH, TOP2A, TSPAN8, EZH2, TAGLN2, LMNB1, PTMA, and TAGLN) that are reported to be differentially expressed at both mRNA and protein level in GBC (Figure 3B; Supplementary Table S7).

### 3.3 Functional enrichment analysis

**FIGURE 4:**
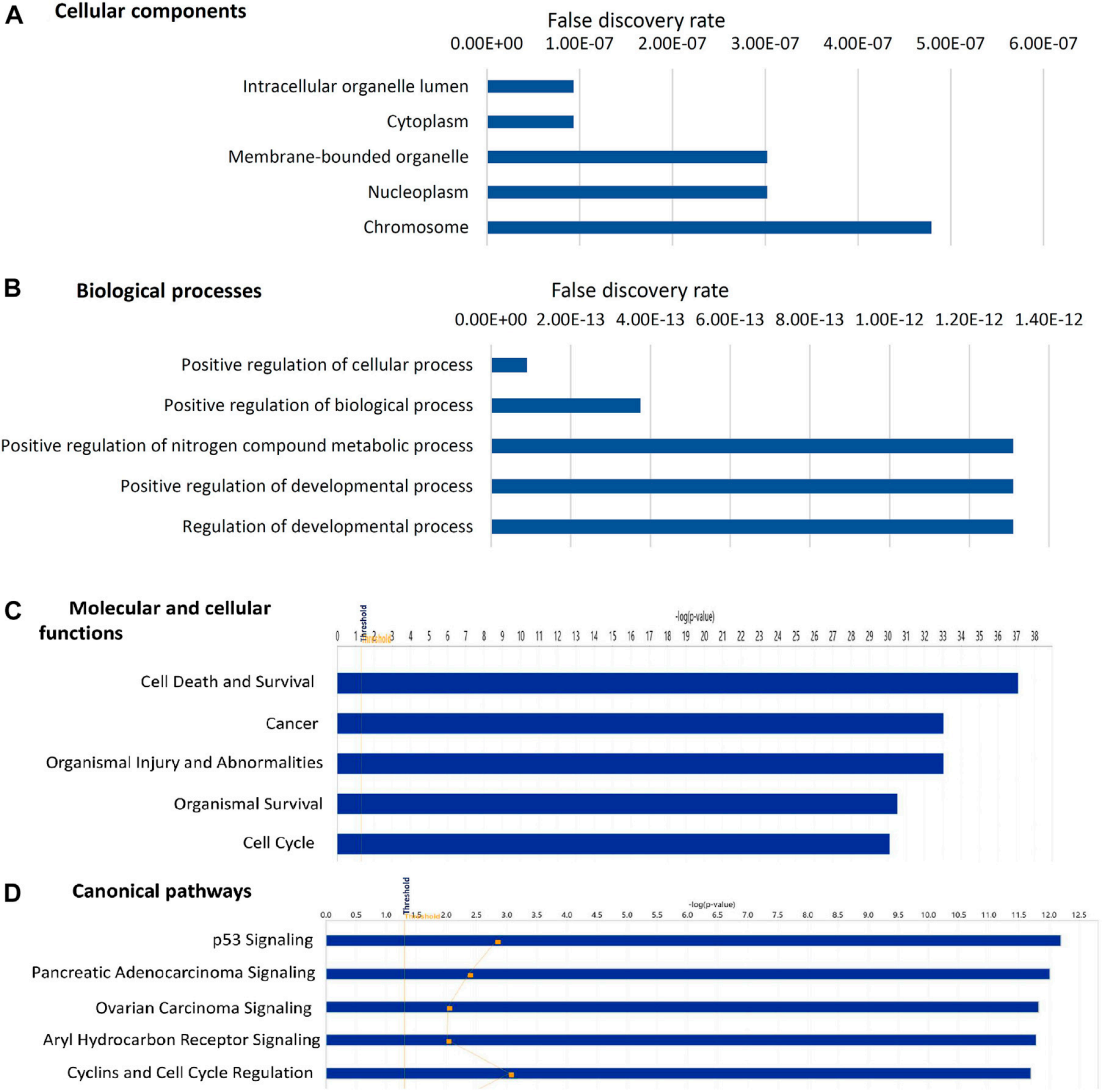
Gene ontology analysis of 183 experimentally observed miRNA targets. The top five cellular components **(A)** biological processes **(B)** molecular and cellular functions **(C)** canonical pathways **(D)** associated with these targets are shown in the figure. The threshold criteria considered for the analysis are -log *p*-value >1.3 or false discovery rate <0.05. The genes associated with canonical pathways are provided in Supplementary Table S5.

Gene ontology analysis of 183 proteins through STRING showed that these are localized in intracellular organelle lumen, cytoplasm, membrane-bound organelle, nucleoplasm and chromosome (Figure 4A). The top biological processes include positive regulation of cellular process, biological process, metabolic process and developmental process (Figure 4B). Molecular and cellular function and canonical pathways were analyzed through IPA and the threshold criteria considered for the analysis are -log *p*-value >1.3 or *p*-value <0.05. The top molecular and cellular functions include cell death and survival, cancer, organismal injury and abnormalities, organismal survival, and cell cycle (Figure 4C). The top canonical pathways include p53 signaling pathway, pancreatic adenocarcinoma signaling, ovarian carcinoma signaling, Aryl hydrocarbon receptor signaling, cyclins and cell cycle regulation (Figure 4D; Supplementary Table S8).

### 3.3 Protein-protein interaction analysis

**FIGURE 5:**
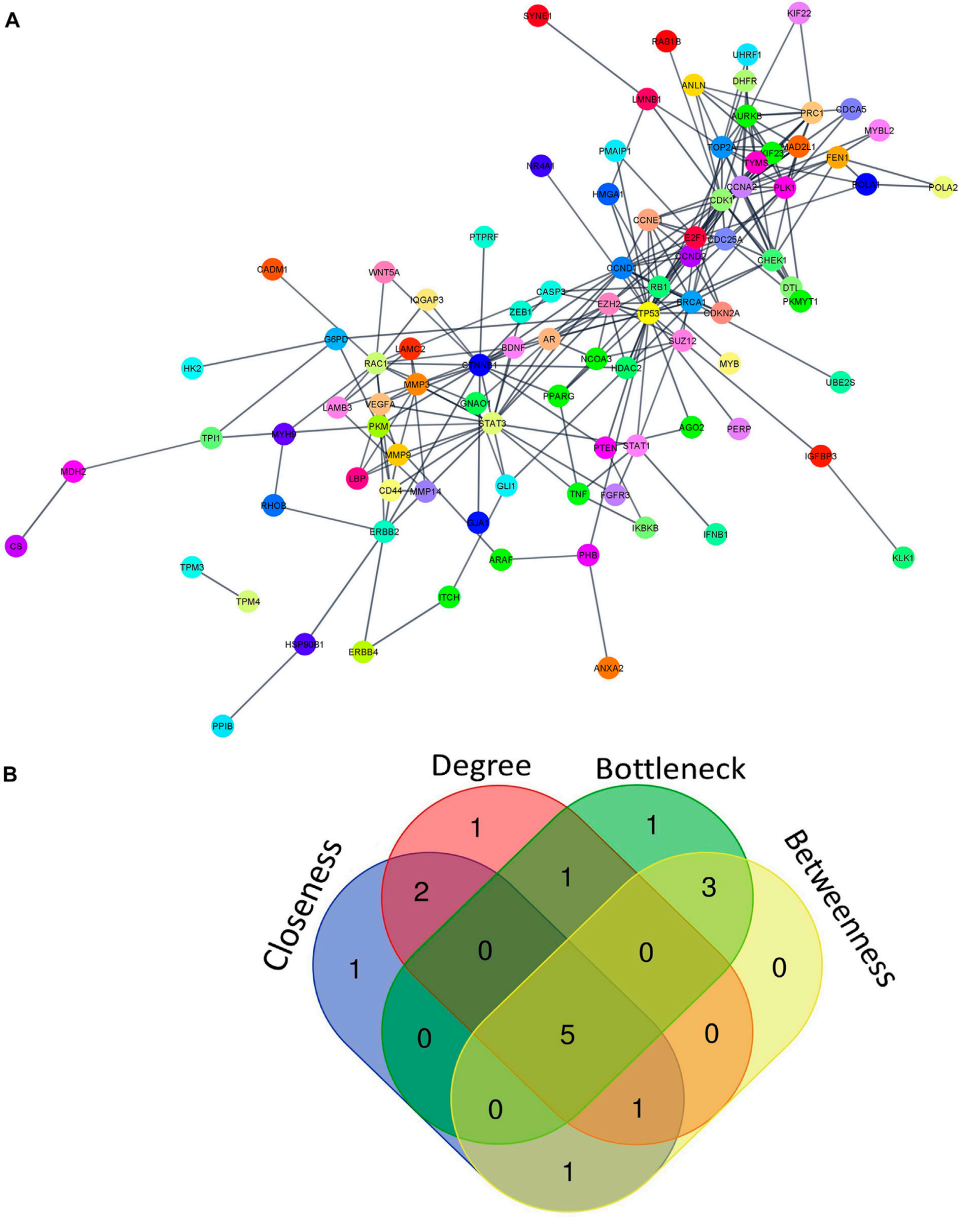
miRNA-regulated protein-protein interaction network. **(A)** PPI network of 183 miRNA targets **(B)** The intersection of the top 10 nodes ranked by degree, betweenness, closeness and bottleneck. We found 5 hub genes including TP53, STAT3, CTNNB1, CDK1, and CCND1. PPI- protein-protein interaction.

PPI analysis of 183 miRNA targets using STRING was visualized by cytoscape (Figure 5A). A total of 5 hub molecules (TP53, STAT3, CTNNB1, CDK1, CCND1) were identified based on the intersection of the top 10 nodes ranked by degree, closeness, betweenness and bottleneck centrality (Figure 5B). Three of the hub molecules (TP53, CCND1, CTNNB1) belong to “p53 signaling pathway” (Figure 3A).

### 3.4 ceRNA regulatory networks

**FIGURE 6:**
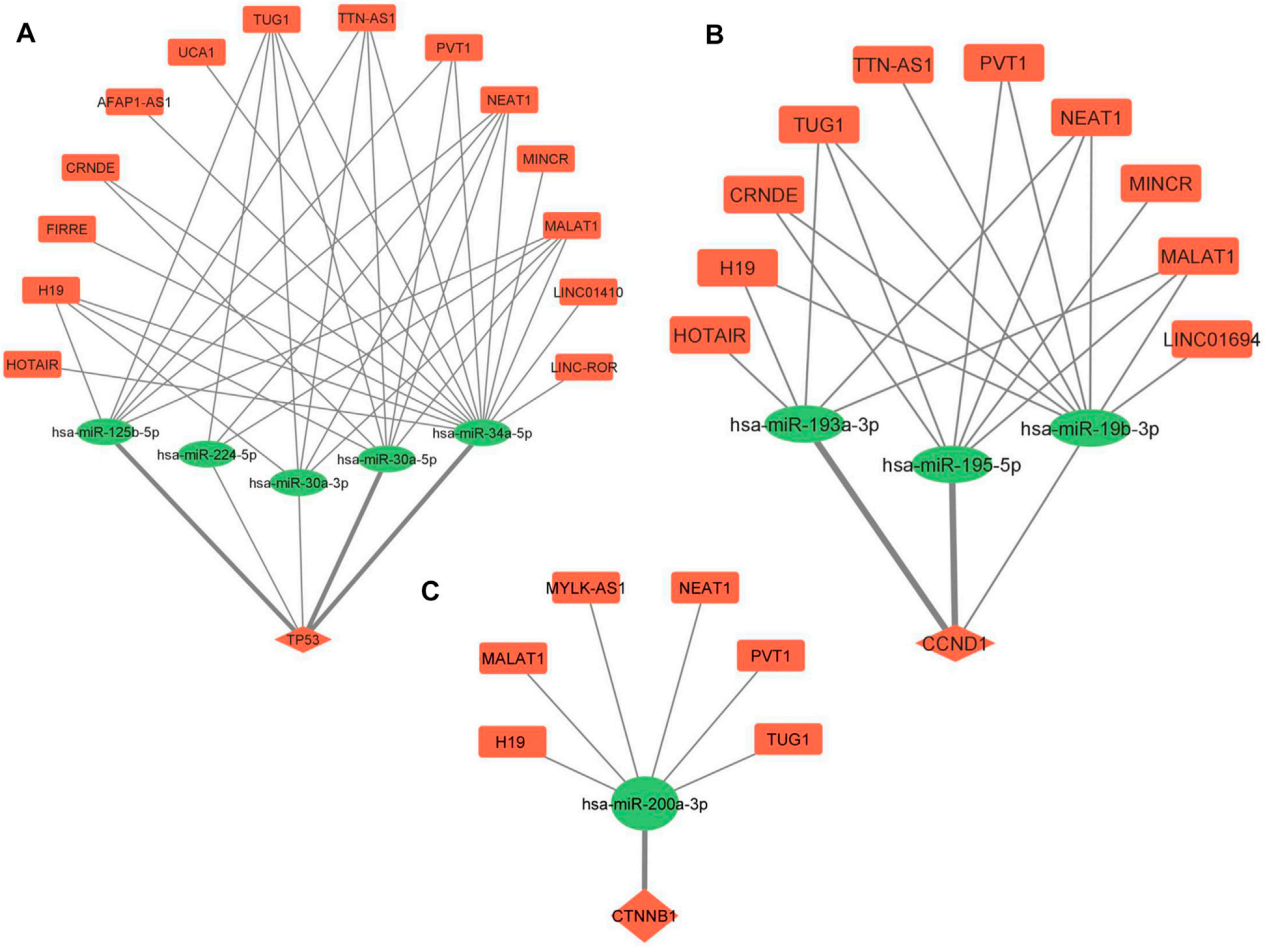
lncRNA-miRNA-mRNA regulatory network of 3 hub molecules among p53 signaling pathway in GBC. **(A)** TP53 **(B)** CCND1 and **(C)** CTNNB1. Different shapes indicate different RNA molecules (Round rectangle‒lncRNA, Ellipse-miRNAs, Diamond-mRNA). The red color indicates “upregulation” and the green color indicates “downregulation” of RNA molecules. mRNA-miRNA interactions with strong evidence, as per the miRTarBase database, are marked with thick lines. GBC, Gallbladder Cancer.

**FIGURE 7:**
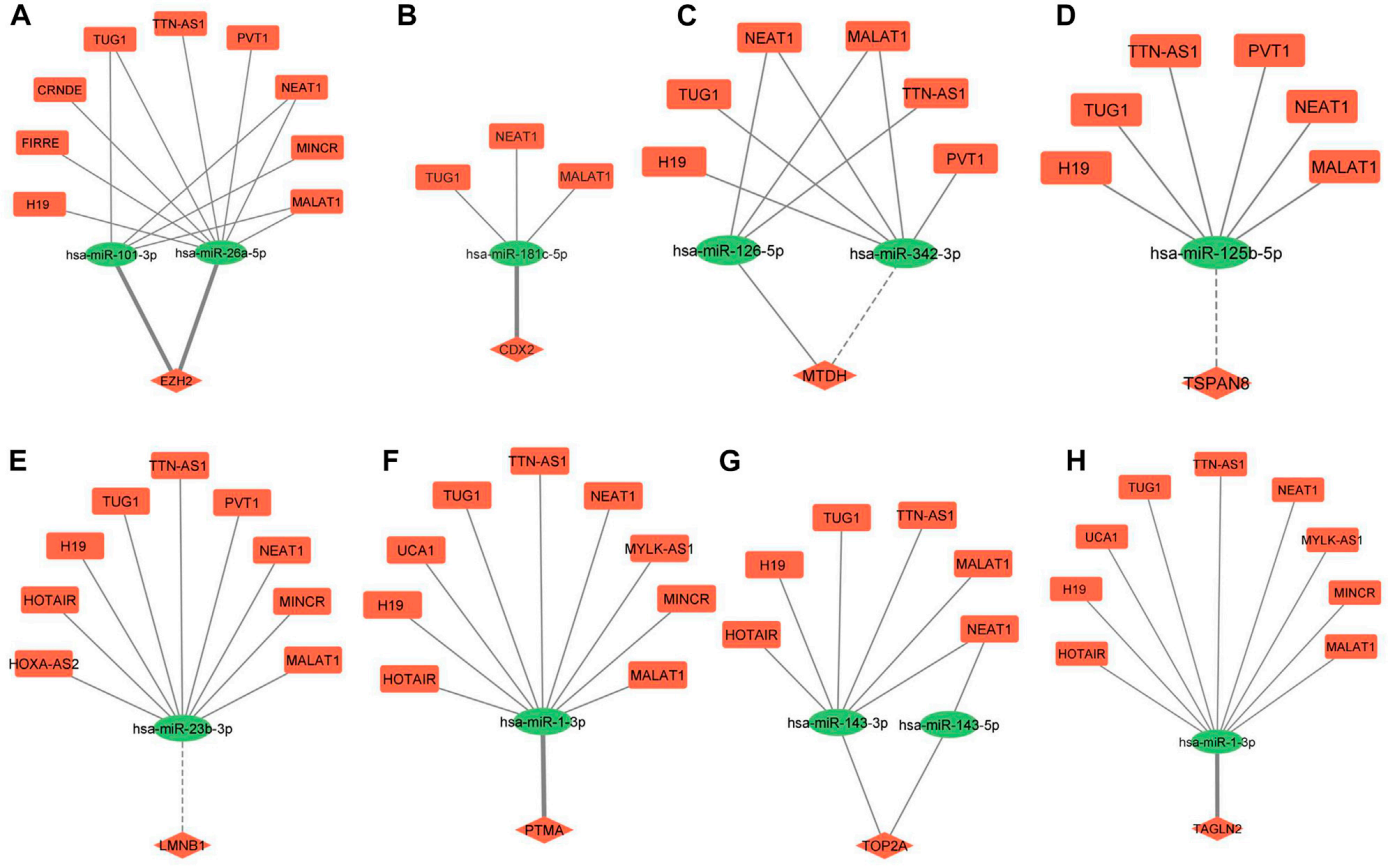
lncRNA-miRNA-mRNA regulatory network of eight miRNA targets DE at both mRNA and protein levels in GBC. **(A)** EZH2 **(B)** CDX2 **(C)** MTDH **(D)** TSPAN8 **(E)** LMNB1 **(F)** PTMA **(G)** TOP2A **(H)** TAGLN2. Different shapes indicate different RNA molecules (Round rectangle‒lncRNA, Ellipse-miRNAs, Diamond-mRNA). The red color indicates “upregulation” and the green color indicates “downregulation” of RNA molecules. mRNA-miRNA interactions with strong evidence are marked with thick lines, and the ones with less strong evidence are marked with dotted lines as per the miRTarBase database. DE, Differentially expressed; GBC, Gallbladder Cancer.

ceRNA regulatory networks were constructed for miRNAs associated with 3 hub molecules (TP53, CTNNB1, CCND1) associated with “p53 signaling pathway”. Since lncRNAs can bind to miRNA and indirectly regulate the translation of targeted mRNAs, the expression of lncRNAs and mRNAs should be positively correlated (López-Urrutia et al., 2019). The lncRNA-miRNAs-mRNA networks for p53, CCND1, and CTNNB1 is shown in Figure 6. The ceRNA regulatory networks for 8 out of 9 miRNA targets (reported to be DE at both mRNA and protein levels) were also constructed. No lncRNA-miRNA interaction was found for one of the targets, TAGLN. The lncRNA-miRNAs-mRNA networks for CDX2, MTDH, TOP2A, TSPAN8, EZH2, TAGLN2, LMNB1, and PTMA is shown in Figure 7. All the lncRNA-miRNA interactions were reported to be strong interactions except for hsa-miR-23b-3p and associated lncRNAs. The miRNA-mRNA interactions with “strong evidence” as per miRTarBase are shown with thick lines and the ones that are with “less strong evidence” is shown with a dashed line (Figures 6, 7).

## 4 Discussion

The molecular mechanism associated with the development and progression of GBC is not clear. An understanding of the ceRNA regulatory networks targeting the “tumor-associated proteins” in GBC would be highly important. In the present study, we integrated the “tissue-based” datasets (lncRNAs, miRNAs, mRNAs and proteins) from high-throughput and/or targeted studies to identify novel ceRNA regulatory networks in GBC. IPA analysis was performed for miRNA-mRNA interactions (predicted and experimentally observed) using DEMs and DEGs or DEPs. We focused on “experimentally observed targets” for associated canonical pathways and PPI analysis to identify hub molecules. In this study, we included the protein dataset to explore the interactions in which the miRNA target is DE at both mRNA and protein levels with a positive correlation in expression in GBC. Then, lncRNA-miRNA-mRNA regulatory networks were constructed for the selected targets, and the following strategy was used for screening the potential ceRNA regulatory network. First, the miRNA-mRNA interactions with strong evidence were screened as per miRTarBase and a literature survey was done for any report on these interactions in cancer conditions. Then, lncRNA-miRNA pairs were also screened for any report of cancer. Further, using the miRNA-mRNA interactions and lncRNA-miRNA interactions, we propose ceRNA networks (lncRNA-miRNA-mRNA) possibly functional in GBC and may have a potential for therapeutic applications in GBC.

We found “p53 signaling pathway” to be among the top pathway associated with 183 miRNA targets. PPI network analysis using these miRNA targets showed 5 hub molecules, 3 of them (p53, CCND1 and CTNNB1) were associated with p53 signaling pathway. TP53, a tumor suppressor gene, affects the cell cycle mechanism and programmed cell death (apoptosis) (Lowe and Lin, 2000; Yildirim et al., 2015). It is reported to be overexpressed in ∼56% of GBC cases (Ghosh et al., 2013) and 70% of GBC cases (Misra et al., 2000). Cyclin D1 (CCND1) participates in the cell cycle phase transition (G1/S phase) (Luo et al., 2017). Accumulation of *β*-catenin promotes the transcription of many oncogenes such as c-Myc and CyclinD-1 (Shang et al., 2017). Both CCND1 and CTNNB1 are negatively regulated by the p53 genes (Liu et al., 2001; Swaminathan et al., 2012).

Further, lncRNA-miRNA-mRNA regulatory networks for p53, CCND1 and CTNNB1 revealed novel ceRNAs possibly regulating the expression of p53 in GBC. We found three p53-miRNAs interactions (miR-125b-5p, miR-34a-5p, and miR-30a-5p), with strong evidence (miRTarBase), regulating the expression of p53 (Figure 6A). Le et al. (2009) highlighted the importance of miR-125b, a brain-enriched miRNA, in the negative regulation of p53 and p53-induced apoptosis during development and stress response. miR-125b has been reported as an oncogene that inhibits cell apoptosis by negatively regulating p53 expression (Wu et al., 2013). There is no report on the regulation of p53 through miR-34a-5p and miR-30a-5p in cancer. In one of our networks (Figure 6B), miR-193a-3p and miR-195-5p are regulating the expression of CCND1, and they are also reported to participate in the pathogenesis of hepatocellular carcinoma and pancreatic ductal adenocarcinoma, respectively, by targeting CCND1 (Chen et al., 2019; Wang et al., 2020b). miRNA-195 inhibits cell proliferation, migration and invasion in epithelial ovarian carcinoma (EOC). In addition, a negative correlation of miR 195 expression with that of CDC42 and CCND1 expression levels was also observed in EOC (Hao et al., 2020). We found miR-200a to be regulating the expression of CTNNB1 in our network (Figure 6C). miR-200a is demonstrated to target CTNNB1 in nasopharyngeal carcinoma (Xia et al., 2010).

Literature search on “lncRNA-miRNA pairs” in cancer revealed one lncRNA (MALAT1) -miR125b interaction in laryngocarcinoma (Zong et al., 2021), three lncRNA (TUG1, NEAT1, MALAT1) -miR-34a interactions reported in endometrial cancer, Nasopharyngeal Cancer, Melanoma (Liu et al., 2017; Ji et al., 2019; Li et al., 2019) and three lncRNA (PVT1, NEAT1, and MALAT1) -miR-30a-5p interactions in papillary thyroid carcinoma, gastric cancer, Hepatocellular Carcinoma (Feng et al., 2018; Pan et al., 2018; Rao et al., 2021). Previous studies revealed that the lncRNAs H19 and NEAT1 were found to directly target miR-193a-3p in Hepatocellular Carcinoma and Lung Adenocarcinoma, respectively (Ma et al., 2018; Xiong et al., 2018). PVT1 is reported to target miR-195 in osteosarcoma (Zhou et al., 2016). H19 has been reported to competitively bind to miR-200a and indirectly regulate *β*-catenin in colorectal cancer (Yang et al., 2017). MALAT1 is found to competitively bind to miR-200a-3p in non-small cell lung cancer (Wei et al., 2019).

Here, six ceRNA regulatory networks (MALAT1-miR125b-p53; PVT1/MALAT1-miR-195-CCND1, H19/NEAT1-miR-193a-3p-CCND1, H19/MALAT1-miR-200a-CTNNB1) were found for which lncRNA-miRNA and/or miRNA-mRNA interactions have been reported in other cancers. In view of the correlation in expression of these lncRNA, miRNA and mRNA, the ceRNA networks appear to play key regulatory roles and may be explored in GBC.

Out of 242, a total of 11 interactions include 9 targets reported to be DE at both mRNA and protein levels in GBC. Out of 9, we found lncRNA-miRNA-mRNA regulatory networks for 8 of them (CDX2, MTDH, TOP2A, TSPAN8, EZH2, TAGLN2, LMNB1, and PTMA) (Figure 7). The same strategy as explained earlier was used for screening the potential ceRNA regulatory networks possibly functional in GBC. We found miRNA-mRNA interaction for a total of 4 genes (EZH2, CDX2, TAGLN2, and PTMA). EZH2 has different roles in cancer, such as oncogenic, tumor suppressor, cancer cell metastasis, cancer immunity, and metabolism. Previous studies have shown its overexpression in different cancers, including prostate cancer, breast cancer, etc. (Duan et al., 2020). PTMA is upregulated and associated with the development of various cancers, including esophageal squamous cell carcinoma, colorectal, bladder, lung, and liver cancer (Zhu et al., 2019). The Caudal-type homeobox transcription factor 2 (CDX2) gene is a specific intestinal transcription factor that is involved in the embryonic development and differentiation of the intestine. Its overexpression in gastric carcinoma cells significantly inhibits cell growth and proliferation (Xie et al., 2010). Transgelin 2 (TAGLN2) is known to bind to actin to facilitate the formation of cytoskeletal structures. Downregulation of transgelin 2 is reported to promote breast cancer metastasis (Yang et al., 2019).

We found miRNA-mRNA interactions (miR-26a-5p/miR-101-3p-EZH2; miR-181c-5p-CDX2; miR1-3p-TAGLN2/PTMA) involving four genes. Interestingly, miR-26a-5p-EZH2 interaction was found to be involved in cell proliferation, cell invasion and apoptosis in GBC cells (Wang et al., 2016b). EZH2 is reported to be a direct target of miR-26a in Uveal Melanoma (UM) cells. Further, the knockout of EZH2 mimicked the tumor inhibition of miR-26a in UM cells (Li et al., 2021). A recent study demonstrated that miR-101-3p prevented retinoblastoma cell proliferation by targeting EZH2 and HDAC9 (Jin et al., 2018), prevented autophagy in endometrial cancer cells by targeting EZH2 (Wang and Liu, 2018) and inhibits invasion and metastasis in renal cell carcinoma by Targeting EZH2 (Dong et al., 2021). PTMA was identified as a target gene regulated by the miR-1 in bladder cancer (Yamasaki et al., 2012).

A literature search on “lncRNA-miRNA pairs” in cancer revealed two lncRNA (NEAT1, MALAT1) -miR-101-3p interactions in lung cancer (Wang et al., 2017b; Kong et al., 2019b), two lncRNA (TUG1, MALAT1) -miR-26a-5p interactions observed in colon cancer and colorectal cancer (Tian et al., 2019; Zhou et al., 2021) and two lncRNA (TUG1, MALAT1) -miR-1-3p interactions in hepatic carcinoma, esophagus cancer (Li et al., 2020; Tang et al., 2022). We found six ceRNA regulatory networks (TUG1/MALAT1-miR-26a-5p-EZH2; NEAT1/MALAT1-miR-101-3p- EZH2; TUG1/MALAT1-miR-1-3p-PTMA) targeting two genes, EZH2 and PTMA. In view of the functional role of EZH2 and PTMA in cancer, as discussed earlier, the ceRNA regulatory network targeting these two genes may be investigated in GBC.

Overall, we identified twelve ceRNA regulatory networks which might be functional in GBC, however, the experimental validation of these networks (expression analysis) in the clinical samples is the limitation of the present study. In future, *in vitro* and *in vivo* studies would be planned that might establish the functional role of these networks in GBC.

## 5 Conclusion

The present study used the experimental data from high-throughput/targeted studies on miRNAs, mRNAs and proteins in GBC and identified 183 miRNA targets. IPA analysis showed “p53 signaling pathway” to be the top pathway associated with them. Three of the targets were among the top 5 hub molecules in PPI network analysis. A total of 9 targets were reported to be DE at both mRNA and protein levels. Further, lncRNA-miRNA-mRNA regulatory networks were constructed for 3 targets (TP53, CCND1, and CTNNB1) associated with p53 signaling pathway and 9 targets (CDX2, MTDH, TOP2A, TSPAN8, EZH2, TAGLN2, LMNB1, and PTMA) DE at both mRNA and protein level. Overall, twelve ceRNA regulatory networks (MALAT1-miR125b-p53; PVT1/MALAT1-miR-195-CCND1, H19/NEAT1-miR-193a-3p-CCND1, H19/MALAT1-miR-200a-CTNNB1, TUG1/MALAT1-miR-26a-5p-EZH2; NEAT1/MALAT1-miR-101-3p- EZH2; TUG1/MALAT1-miR-1-3p-PTMA) were identified for which lncRNA-miRNA and/or miRNA-mRNA interactions have been reported in other cancers. These and other lncRNA-miRNA-mRNA regulatory networks may be experimentally validated and explored for their therapeutic applications in GBC.

## Data Availability

The original contributions presented in the study are included in the article/Supplementary Material, further inquiries can be directed to the corresponding author.
